# Selection of Reference Genes for Expression Analysis Using Quantitative Real-Time PCR in the Pea Aphid, *Acyrthosiphon pisum* (Harris) (Hemiptera, Aphidiae)

**DOI:** 10.1371/journal.pone.0110454

**Published:** 2014-11-25

**Authors:** Chunxiao Yang, Huipeng Pan, Yong Liu, Xuguo Zhou

**Affiliations:** 1 Hunan Academy of Agricultural Sciences, Institute of Plant Protection, Changsha, Hunan, China; 2 Department of Entomology, University of Kentucky, Lexington, Kentucky, United States of America; Wuhan Bioengineering Institute, China

## Abstract

To facilitate gene expression study and obtain accurate qRT-PCR analysis, normalization relative to stable expressed housekeeping genes is required. In this study, expression profiles of 11 candidate reference genes, including *actin (Actin)*, *elongation factor 1 α (EF1A)*, *TATA-box-binding protein (TATA)*, *ribosomal protein L12 (RPL12)*, *β-tubulin (Tubulin)*, *NADH dehydrogenase (NADH)*, *vacuolar-type H+-ATPase (v-ATPase)*, *succinate dehydrogenase B (SDHB)*, *28S ribosomal RNA (28S)*, *16S ribosomal RNA (16S)*, and *18S ribosomal RNA (18S)* from the pea aphid *Acyrthosiphon pisum*, under different developmental stages and temperature conditions, were investigated. A total of four analytical tools, *geNorm*, *Normfinder*, *BestKeeper*, and the *ΔC_t_* method, were used to evaluate the suitability of these genes as endogenous controls. According to *RefFinder*, a web-based software tool which integrates all four above-mentioned algorithms to compare and rank the reference genes, *SDHB, 16S, and NADH* were the three most stable house-keeping genes under different developmental stages and temperatures. This work is intended to establish a standardized qRT-PCR protocol in pea aphid and serves as a starting point for the genomics and functional genomics research in this emerging insect model.

## Introduction

Quantitative real-time PCR (qRT-PCR) is a rapid and reliable method for the detection and quantification of gene expression levels during different biological processes [1]. Although qRT-PCR is often described as the gold standard, there are still some limitations of this assay such as RNA quality and quantity, reverse transcription and normalization, and efficiency of PCR reaction can influence threshold cycle (C_t_)values [2], [3]. A common technique in qRT-PCR is to normalize data by measuring in parallel the expression of a reference gene from the same samples. Using housekeeping genes as a reference is the most widely adopted approach [1]. Housekeeping genes are believed to possess inherent stable and constitutive expression irrespective of physiological conditions in different samples or treatments under investigation [4], [5]. Several reports have demonstrated that some commonly used reference genes differentially expressed under different treatments or conditions [4]–[8]. In fact, no reference genes are stably expressed and suitable for the entire cell and tissue, and various experimental conditions [4]–[8].

The pea aphid, *Acyrthosiphon pisum* (Harris) (Hemiptera, Aphidiae), is an important cosmopolitan pest. It feeds on a wide range of legume plants(family *Fabaceae*) worldwide, including pea, clover, alfalfa, and broad bean, and is considered as the aphid species of major agronomical importance [9]. More importantly, it can transmit over 30plant viruses [10]. In addition, *A. pisum*is an emerging model organism for the studies of insect-plant interactions, especially after the release of its genome in 2010 [11]. With the advent of omics tools, there is an unprecedented opportunity to investigate the genetic basis of its physiological and biological functions [12], [13]. There have been demonstrated needs for the systematic validation of references genes in qRT-PCR analysis, normalization procedures have yet received any attention for this emerging insect model.

The objective of this study was to address an important aspect of gene expression studies in the pea aphid, *Acyrthosiphon pisum*, as well as in other insects which is the selection of appropriate references genes with stable expression under different experimental conditions. Here, the expression profiles of 11 candidate reference genes, including *actin (Actin)*, *elongation factor 1 α (EF1A)*, *TATA-box-binding protein (TATA)*, *ribosomal protein L12 (RPL12)*, *β-tubulin (Tubulin)*, *NADH dehydrogenase (NADH)*, *vacuolar-type H+-ATPase (v-ATPase)*, *succinate dehydrogenase B (SDHB)*, *28S ribosomal RNA (28S)*, *16S ribosomal RNA (16S)*, and *18S ribosomal RNA (18S)* from the pea aphid genome [11], were examined under different developmental stages and temperatures. As a result, different sets of reference genes were recommended accordingly.

## Materials and Methods

### Insects

Pea aphid, *Acyrthosiphon pisum* (Harris) (Hemiptera, Aphidiae)colony was kindly provided by Dr. John Obrycki (University of Kentucky). Aphids were maintained at 20–28°C on seedlings of fava bean, *Viciafaba* (Fabales, Fabaceae)in a greenhouse.

### Samples preparation

Fifteen adult females were allowed to lay the offspring for 24 h on fava bean leaves resting on wet filter paper in a petri dish (9 cm diameter). Then10 adults as one replicate and 20nymphs (less than 24 h old) as one replicate, respectively were exposed to 10°C, 22°C, and 30°C, respectively for 2 d in a climate chamber with a photoperiod of 14: 10 (L: D) and 50% relative humidity. All collected samples were preserved in 1.5 ml centrifuge tubes and stored at −80°C after being frozen in liquid nitrogen. Each treatment was repeated three times independently, therefore, there are 18 biological samples in total.

### Total RNA extraction and cDNA synthesis

Total RNA was extracted using TRIzol (Invitrogen, Carlsbad, CA) following the manufacturer's instruction. First-strand cDNA was synthesized from 1 µg of total RNA using the M-MLV reverse transcription kit (Invitrogen, Carlsbad, CA) according the manufacturer's recommendations.

### Reference gene selection and primer design

Eleven commonly used reference genes were selected (Table 1). PCR amplifications were performed in 50 µl reactions containing 10 µl 5×PCR Buffer (Mg^2+^ Plus), 1 µldNTP mix (10 mM of each nucleotide), 5 µl of each primer (10µM each), and 0.25 µl of Go Taq(5 u/µl) (Promega). The PCR parameters were as follows: one cycle of 94°C for 3 min; 35 cycles of 94°C for 30 s, 59°C for 45 s, and 72°C for 1 min; a final cycle of 72°C for 10 min. Amplicons of the expected size were purified and cloned into the pCR4-TOPO vector (Invitrogen, Carlsbad, CA) for sequencing confirmation.

**Table 1:** Primer sets for qRT-PCR analysis.

Gene	Accession No.	Primer sequences (5′-3′)	Length (bp)	E (%)*	R^2,^**
*EF1A*	AY219737	F: AGAATGGACAAACCCGTGAA	104	104.8	0.9989
		R: GCTGTATGGTGGTTCAGTAGAG			
*Tublin*	NM_001190398	F: TTGGTACACTGGTGAAGGTATG	103	103.5	0.9980
		R: AGCGGTAGCTTCTTGGTATTG			
*NADH*	NM_001162323	F: CGAGGAGAACATGCTCTTAGAC	93	104.6	0.9989
		R:GATAGCTTGGGCTGGACATATAG			
*RPL12*	NM_001126171	F: AAGGCTACATCTGACTGGAAAG	101	108.0	0.9992
		R: ACCAATGATGATGCAGAAGGA			
*SDHB*	NM_001162436	F: CTGAATTCCTGTGGACCTATGG	90	102.9	0.9963
		R: ACGGCAAGAACGCCTAAA			
*18S*	X62623	F: CCGCGAAACCGTCATTAAATC	101	103.9	0.9921
		R: GGAACTCTGTCGGCATGTATTA			
*28S*	S50426	F: CGGGTGGTAAACTCCATCTAAC	118	97.2	0.9943
		R:CGAGCGGTTTCACGTTCTTA			
*16S*	FJ411411	F:AGAAACCAACCTGGCTTACAC	121	109.9	0.9989
		R:TTGCGACCTCGATGTTGAATTA			
*v-ATPAase*	NM_001126156	F: CTTCTCTGCTGAGTGCTGTT	90	99.4	0.9931
		R: GCCATCACGACGACTGATTA			
*Actin*	NM_001126200	F: CGTTACCAACTGGGACGATATG	111	105.8	0.9985
		R: GGGTTCAATGGAGCTTCTGTTA			
*TATA*	NM_001162717	F: CACCTAATGTCACCAGCCTATT	123	106.9	0.9992
		R: TGTGTCCAAGGCGTTCTAAG			

“*”: PCR efficiency (calculated from the standard curve).

“**”: Regression coefficient.

### Quantitative real-time PCR

Gene-specific primers (Table 1) were used in PCR reactions (20 µl) containing 7µl of ddH_2_O, 10 µl of 2×SYBR Green MasterMix (Bio-Rad), 1 µl of each specific primer (10 µM), and 1 µl of first-strand cDNA template. The qPCR program included an initial denaturation for 3 min at 95°C followed by 40 cycles of denaturation at 95°C for 10 s, annealing for 30 s at 55°C, and extension for 30 s at 72°C. For melting curve analysis, a dissociation step cycle (55°C for 10 s, and then 0.5°C for 10 s until 95°C) was added. The reactions were set up in 96-well format Microseal PCR plates (Bio-Rad) in triplicates. All experiments were replicated in triplicate.

Reactions were performed in a MyiQ single Color Real-Time PCR Detection System (Bio-Rad). Existence of single peaks in melting curve analysis was used to confirm gene-specific amplification and rule out non-specific amplification and primer-dimer generation. The qRT-PCR was determined for each gene using slope analysis with a linear regression model. Relative standard curves for the transcripts were generated with serial dilutions of cDNA (1/5, 1/25, 1/125, 1/625, and 1/3125). The corresponding qRT-PCR efficiencies (E) were calculated according to the equation: E = (10^[-1/slope]^ -1)×100.

### Stability of gene expression

All biological replicates were used to calculate the average Ct value. The stability of the ten housekeeping genes were evaluated by algorithms *geNorm* [1], *NormFinder* [14], *BestKeeper* [15], and the comparative *ΔCt* method [16]. Finally, we compared and ranked the tested candidates based on a web-based analysis tool *RefFinder* (http://www.leonxie.com/referencegene.php).

## Results

### Transcriptional profiling of candidate reference genes

First, 11 candidate reference genes were investigated by reverse transcription polymerase chain reaction (RT-PCR). All genes tested were expressed in pea aphid, and visualized as a single amplicon with expected size on a 1.5% agarose gel. All amplicons were sequenced and displayed 100% identity with their corresponding sequences. Furthermore, gene-specific amplification of these genes was confirmed by a single peak in real-time melting-curve analysis. A standard curve was generated for each gene, using five-fold serial dilution of the pooled cDNAs. The correlation coefficient and PCR efficiency for each standard curve were shown in Table 1.

**Figure 1:**
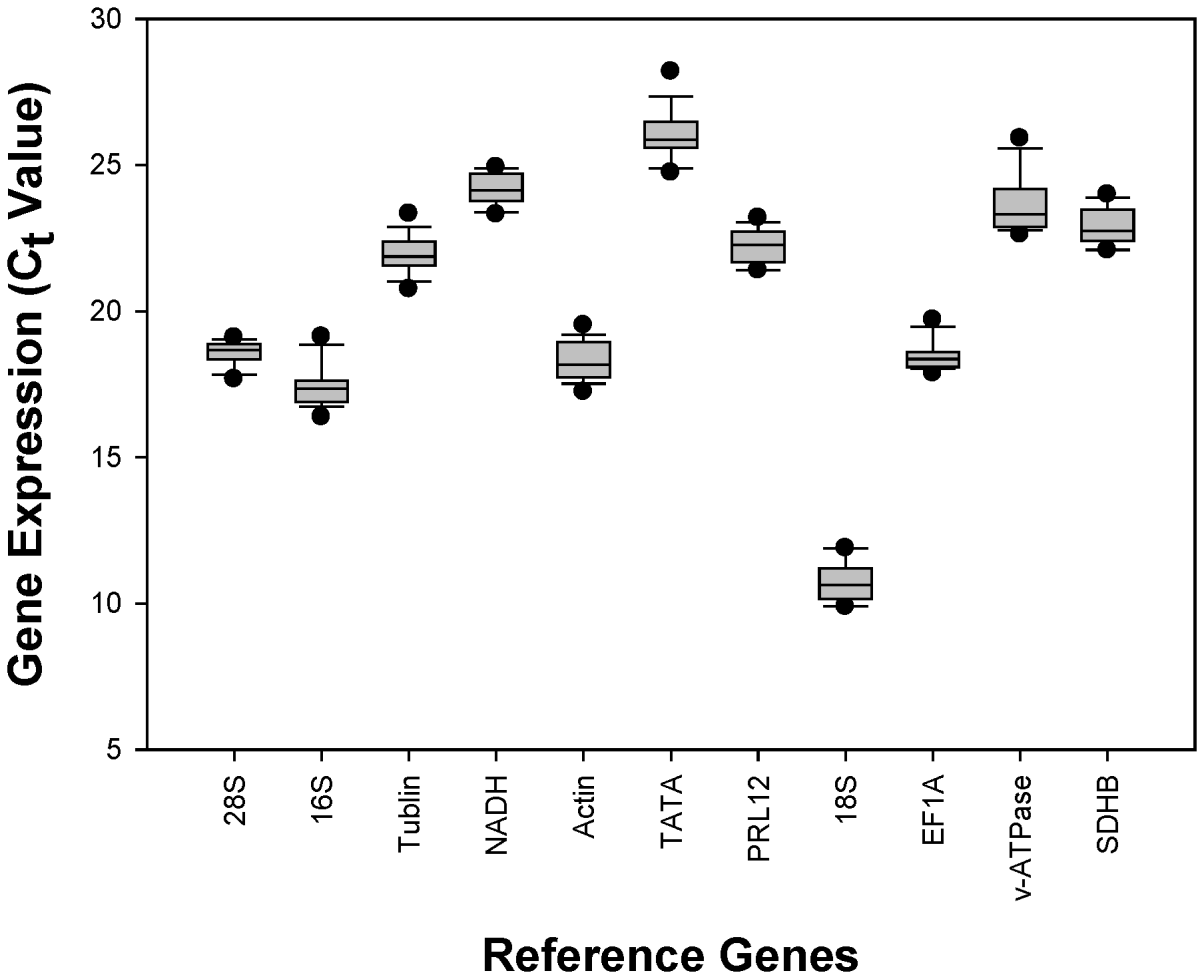
Expression profiles of candidate reference genes in the pea aphid, *Acyrthosiphon pisum*. The expression level of candidate reference genes are documented in C_t_-value. The median is represented by the line in the box. The interquartile rang is bordered by the upper and lower edges, which indicate the 75^th^ and 25^th^ percentiles, respectively.

We calculated the mean and the standard derivation (SD) of the C_t_ values for all the samples together. *v-ATPase*had the most variable expression levels reflected in its high SD values. On the contrary, *28S* had the least variable expression levels reflected in its low SD values. In addition, *TATA* (C_tavg_ = 26.05) had the highest C_t_ values and was therefore the least expressed among the gene candidates. *18S* (C_tavg_ = 10.72) had the lowest C_t_ values and was therefore the mostly expressed among the gene candidates. (Figure 1).

### Quantitative analysis of reference candidates based on *geNorm*

**Figure 2:**
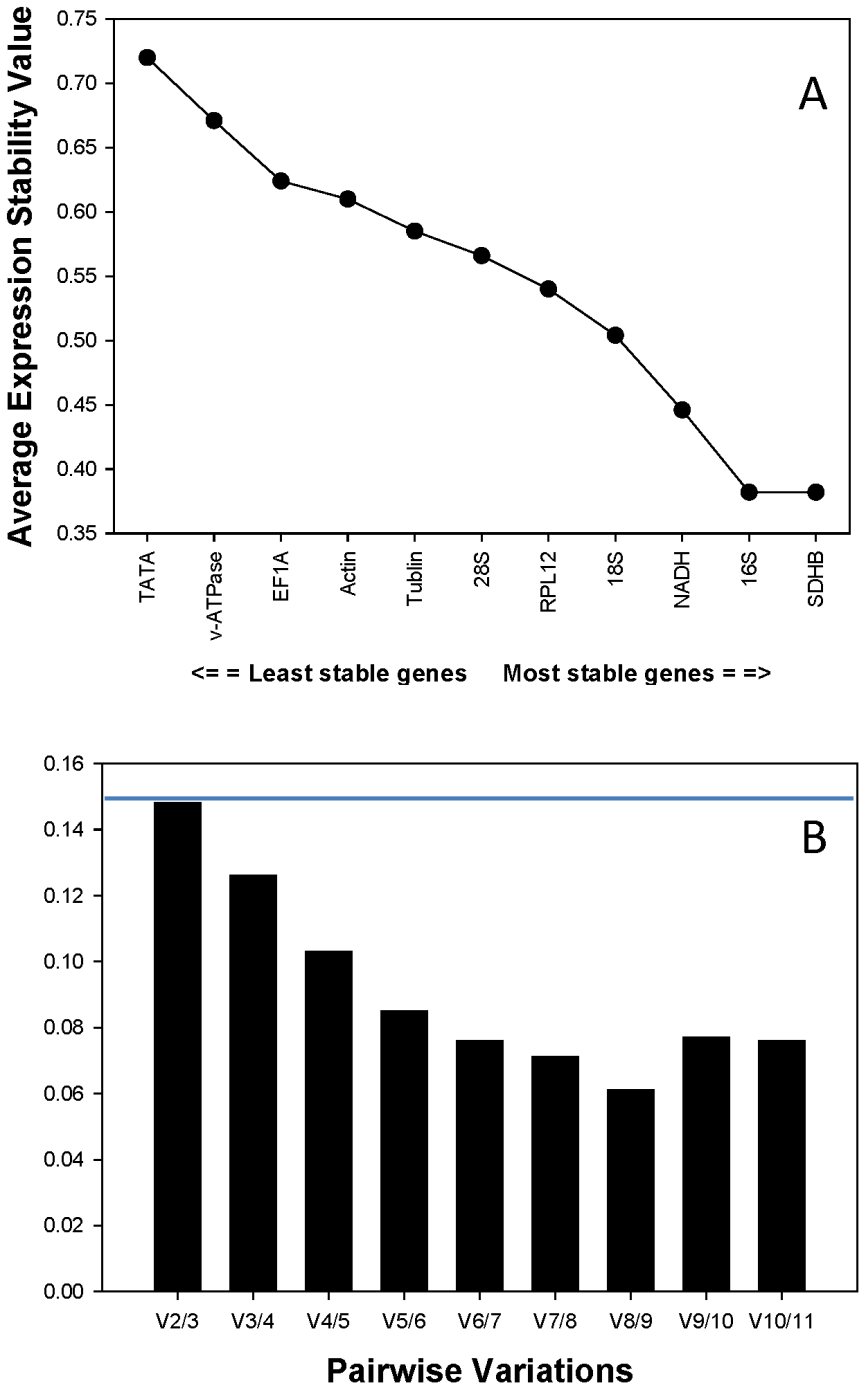
Quantitative and qualitative analysis based on *geNorm*. (A) Ranking of the 11 housekeeping genes based on the stability value (M). A lower stability value indicates more stable expression. (B)Pairwise variation (V) analysis of the candidate reference genes. The *geNorm* first calculates an expression stability value (M) for each gene and then compares the pair-wise variation (V) of this gene with the others. A threshold of V<0.15 was suggested for valid normalization. *geNorm* starts by a gene pair, and tests whether the inclusion of a 3^rd^ gene adds significant variation. The pair-wise variation (V_n_/V_n+1_) was analyzed between the normalization factors NF_n_ and NF_n+1_ by the *geNorm* software to determine the optimal number of references genes required for qRT-PCR data normalization.

To determine the minimal number of genes required for normalization, we computed the V-value by *geNorm*. Starting with two genes, the software sequentially adds another gene and recalculates the normalization factor ratio. If the added gene does not increase the normalization factor ratio above the proposed 0.15 cut-off value, then the original pair of genes is enough for normalization. However, if the new ratio is above 0.15, then more genes should be included. The first V-value <0.15 was after V2/3 (Figure 2B). This means that two reference genes were enough for reliable normalization under the developmental stages and temperature conditions.

### Determining the best reference candidates based on *geNorm*

*GeNorm* bases its ranking on the geometric mean of the SD of each transformed gene set of pair combinations (M-value). The lower the M-value is, the higher the ranking. *SDHB* and *16S* were co-ranked as the most stable genes (M = 0.382). The overall order based on *geNorm* from most stable to least stable reference genes was: *SDHB* = *16S*, *NADH*,*18S*, *RPL12*,*28S*, *Tublin*, *Actin*, *EF1A*, *v-ATPase*, *TATA* (Figure 2A, Table 2).

**Table 2:** Ranking of reference gene candidates using different algorithms*.

*RefFinder*		*geNorm*		*NormFider*		*ΔCt*			*BestKeeper*		
Genes	GM	Genes	SV	Genes	SV	Genes	SV	Genes	[r]	Genes	SD
*SDHB*	1.495	*SDHB*	0.382	*SDHB*	0.194	*SDHB*	0.57	*SDHB*	0.931	*28S*	0.321
*16S*	2.449	*16S*	0.382	*NADH*	0.378	*NADH*	0.64	*16S*	0.852	*EF1A*	0.371
*NADH*	2.449	*NADH*	0.446	*16S*	0.413	*16S*	0.65	*18S*	0.800	*NADH*	0.467
*28S*	4.427	*18S*	0.504	*RPL12*	0.426	*RPL12*	0.67	*v-ATPase*	0.781	*16S*	0.492
*RPL12*	4.681	*RPL12*	0.540	*18S*	0.443	*18S*	0.68	*PRL12*	0.742	*SDHB*	0.492
*18S*	5.318	*28S*	0.566	*Actin*	0.488	*Actin*	0.71	*Actin*	0.715	*PRL12*	0.504
*EF1A*	5.450	*Tublin*	0.585	*EF1A*	0.503	*EF1A*	0.71	*NADH*	0.680	*Tublin*	0.529
*Actin*	7.135	*Actin*	0.610	*28S*	0.527	*28S*	0.73	*Tublin*	0.606	*18S*	0.540
*Tublin*	7.937	*EF1A*	0.624	*Tublin*	0.546	*Tublin*	0.74	*EF1A*	0.442	*Actin*	0.590
*v-ATPase*	10.241	*v-ATPase*	0.671	*v-ATPase*	0.774	*v-ATPase*	0.89	*28S*	0.350	*TATA*	0.592
*TATA*	10.741	*TATA*	0.720	*TATA*	0.820	*TATA*	0.94	*TATA*	0.330	*v-ATPase*	0.802

“*”: Geometric mean (GM); Stability Value (SV); Pearson's correlation coefficient ([r]); Standard Deviation (SD).

### Determining the best reference candidates based on *ΔC_t_* method

Gene ranking using the *ΔC_t_* method relies on relative pair-wise comparisons. Using raw *C_t_* values, the average SD of each gene set is inversely proportional to gene stability. As shown in Tables S1 and 5, *SDHB* (0.57) was the top-ranked gene. The overall order from most stable to least stable reference genes based on the *ΔC_t_* method was: *SDHB*, *NADH*,*16S*, *RPL12*,*18S*, *Actin*, *EF1A*,*28S*, *Tublin*, *v-ATPase*, *TATA*(Table 2).

### Determining the best reference candidates based on *NormFinder*

*SDHB* (0.194) was the gene with the least variation in expression levels; thus *SDHB* would be the most reliable reference gene. The overall order from most stable to least stable reference genes based on *NormFinder* was: *SDHB*, *NADH*,*16S*, *RPL12*,*18S*, *Actin*, *EF1A*,*28S*, *Tublin*, *v-ATPase*, *TATA* (Table 2).

### Determining the best reference candidates based on *BestKeeper*

*BestKeeper* provided a two-way ranking: Pearson's correlation coefficient and *BestKeeper* computed SD values. The stability of a gene is directly proportional to the [r] value, while it is inversely proportional to the SD value. *SDHB*(r = 0.931) and *16S* (r = 0.852) had the highest[r] value, whereas *28S*(SD = 0.321) and *EF1A* (SD = 0.371) had the least variable expression levels across all the samples (Table S2, 2).

### Comprehensive ranking of best reference genes using *RefFinder*

All software programs except the SD value based on *BestKeeper* indentified *SDHB* as the most stable gene (Table S2). According to *RefFinder*, the overall order from the most stable to the least stable reference genes was:*SDHB*,*16S*, *NADH*,*28S*, *RPL12*,*18S*, *EF1A*, *Actin*, *Tublin*, *v-ATPase*, *TATA*. Among them, *v-ATPAase* and *TATA* both had GM values higher than 10.0 (Table 2), these two candidates had the lowest ranking and less suitable to serve as reliable reference genes for normalizing gene expression.

## Discussion

qRT-PCR quantification requires robust normalization by reference genes to offset confounding variations in experimental data. Most gene expression studies in the literature use a single endogenous control; this will profoundly influence the statistical outcome and may lead to inaccurate data interpretation [17]. Currently, the reference genes studies of many insects have been accomplished including whitefly, diamondback moth, brown planthopper, beet armyworm, oriental leafworm moth, Colorado potato beetle, and oriental fruit fly [4]–[8], [18], [19]. Here, the expression profiles of 11 candidate reference genes from the pea aphid were evaluated under different developmental stages and temperature conditions. Our finding is the first step toward establishing a standardized qRT-PCR analysis for this research model.

*BestKeeper* ranked the genes different from the other analysis methods used (Table 2). Unlike *Genorm* and *NormFinder*, *BestKeeper* is not specifically built to construct a hierarchy of reference genes. Instead, *BestKeeper* is intent to establish the best possible referencing point using an averaged expression of multiple housekeeping genes. Therefore, based on the needs, different analytical tools should be considered. There has been ongoing discussion about the optimal number of reference genes required for qRT-PCR analysis. The fact is that multiple reference genes are increasingly used to analyze gene expression under various experimental conditions in a given experiment, because one reference gene is usually insufficient to normalize the expression results of target genes [20]. This can decreased the probability of biased normalization. Our results demonstrated that the use of two reference genes can be sufficient to normalize the expression data and provides amore conservative estimation of target gene expression (Figure 2). As a result, we strongly suggest that two internal references are necessary for studying gene expression in pea aphid under different developmental stages and temperature conditions.

This is the first study to evaluate candidate reference genes for gene expression analyses in the pea aphid. Based on the comprehensive analysis, *SDHB, 16S, and NADH* were the three most stable house-keeping genes under different developmental stages and temperature conditions. This study not only sheds light on establishing a standardized qRT-PCR procedure in pea aphid, but also lays a solid foundation for the genomics and functional genomics research in this insect.

## Supporting Information

Table S1 **Summary of mean and SD values of gene pairwise comparison using the** ***ΔCt*** **method for 11 gene candidates.**
(DOCX)

Table S2 **Ranking of 11 reference gene candidates based on** ***BestKeeper*** **.** Two criteria are considered: Pearson's correlation coefficient and *BestKeeper* computed SD values. The stability of a gene is directly proportional to the [r] value, while it is inversely proportional to the SD value.
(DOCX)
