# Supplementary material for: Selection of Reference Genes for Expression Analysis Using Quantitative Real-Time PCR in the Pea Aphid, Acyrthosiphon pisum (Harris) (Hemiptera, Aphidiae)
Source: PLoS One. 2014 Nov 25;9(11):e110454. doi: 10.1371/journal.pone.0110454 (PMC4244036; doi:10.1371/journal.pone.0110454)
Supplement: Table S1 — Summary of mean and SD values of gene pairwise comparison using the ΔCt method for 11 gene candidates. (DOCX) [file pone.0110454.s001.docx]

Table S1. Summary of mean and SD values of gene pairwise comparison using the *ΔCt* method for 11 gene candidates.

| *Gene* |  | Pair 1 | Pair 2 | Pair 3 | Pair 4 | Pair 5 | Pair 6 | Pair 7 | Pair 8 | Pair 9 | Pair 10 | Average SD |
| --- | --- | --- | --- | --- | --- | --- | --- | --- | --- | --- | --- | --- |
| *28S* | Mean | -1.15 | 3.38 | 5.61 | -0.26 | 7.47 | 3.65 | -7.85 | -0.09 | 5.10 | 4.34 |  |
|  | SD | 0.65 | 0.70 | 0.68 | 0.68 | 1.04 | 0.50 | 0.62 | 0.68 | 1.07 | 0.64 | 0.73 |
| *16S* | Mean | 1.15 | 4.53 | 6.76 | 0.89 | 8.62 | 4.80 | -6.70 | 1.06 | 6.25 | 5.49 |  |
|  | SD | 0.65 | 0.60 | 0.55 | 0.78 | 1.06 | 0.63 | 0.51 | 0.83 | 0.53 | 0.38 | 0.65 |
| *Tublin* | Mean | -3.38 | -4.53 | 2.23 | -3.64 | 4.10 | 0.27 | -11.23 | -3.47 | 1.73 | 0.96 |  |
|  | SD | 0.70 | 0.60 | 0.39 | 0.83 | 1.08 | 0.79 | 0.74 | 0.75 | 0.91 | 0.58 | 0.74 |
| *NADH* | Mean | -5.61 | -6.76 | -2.23 | -5.87 | 1.86 | -1.96 | -13.46 | -5.70 | -0.51 | -1.27 |  |
|  | SD | 0.68 | 0.55 | 0.39 | 0.69 | 0.91 | 0.74 | 0.68 | 0.59 | 0.79 | 0.40 | 0.64 |
| *Actin* | Mean | 0.26 | -0.89 | 3.64 | 5.87 | 7.73 | 3.91 | -7.59 | 0.17 | 5.36 | 4.60 |  |
|  | SD | 0.68 | 0.78 | 0.83 | 0.69 | 0.69 | 0.53 | 0.71 | 0.51 | 1.06 | 0.58 | 0.71 |
| *TATA* | Mean | -7.47 | -8.62 | -4.10 | -1.86 | -7.73 | -3.82 | -15.33 | -7.57 | -2.37 | -3.14 |  |
|  | SD | 1.04 | 1.06 | 1.08 | 0.91 | 0.69 | 0.87 | 1.01 | 0.63 | 1.21 | 0.89 | 0.94 |
| *RPL12* | Mean | -3.65 | -4.80 | -0.27 | 1.96 | -3.91 | 2.82 | -11.50 | -3.74 | 1.45 | 0.69 |  |
|  | SD | 0.50 | 0.63 | 0.79 | 0.74 | 0.53 | 0.87 | 0.46 | 0.62 | 0.98 | 0.55 | 0.67 |
| *18S* | Mean | 7.85 | 6.70 | 11.23 | 13.46 | 7.59 | 15.33 | 11.50 | 7.76 | 12.95 | 12.19 |  |
|  | SD | 0.62 | 0.51 | 0.74 | 0.68 | 0.71 | 1.01 | 0.46 | 0.82 | 0.72 | 0.49 | 0.68 |
| *EF1A* | Mean | 0.09 | -1.06 | 3.47 | 5.70 | -0.17 | 7.57 | 3.74 | -7.76 | 5.20 | 4.43 |  |
|  | SD | 0.68 | 0.83 | 0.75 | 0.59 | 0.51 | 0.63 | 0.62 | 0.82 | 1.09 | 0.59 | 0.71 |
| *v-ATPase* | Mean | -5.10 | -6.25 | -1.73 | 0.51 | -5.36 | 2.37 | -1.45 | -12.95 | -5.20 | -0.77 |  |
|  | SD | 1.07 | 0.53 | 0.91 | 0.79 | 1.06 | 1.21 | 0.98 | 0.72 | 1.09 | 0.57 | 0.89 |
| *SDHB* | Mean | -4.34 | -5.49 | -0.96 | 1.27 | -4.60 | 3.14 | -0.69 | -12.19 | -4.43 | 0.77 |  |
|  | SD | 0.64 | 0.38 | 0.58 | 0.40 | 0.58 | 0.89 | 0.55 | 0.49 | 0.59 | 0.57 | 0.57 |
