# Supplementary material for: Selection of Reference Genes for Expression Analysis Using Quantitative Real-Time PCR in the Pea Aphid, Acyrthosiphon pisum (Harris) (Hemiptera, Aphidiae)
Source: PLoS One. 2014 Nov 25;9(11):e110454. doi: 10.1371/journal.pone.0110454 (PMC4244036; doi:10.1371/journal.pone.0110454)
Supplement: Table S2 — Ranking of 11 reference gene candidates based on BestKeeper . Two criteria are considered: Pearson's correlation coefficient and BestKeeper computed SD values. The stability of a gene is directly proportional to the [r] value, while it is inversely proportional to the SD value. (DOCX) [file pone.0110454.s002.docx]

Table S2. Ranking of 11 reference gene candidates based on *BestKeeper*. Two criteria are considered: Pearson’s correlation coefficient and *BestKeeper* computed SD values. The stability of a gene is directly proportional to the [r] value, while it is inversely proportional to the SD value.

| Gene | GM  [C_t_] | AM | Min | Max | SD | CV | [r] | p-value | Ranking | |
| --- | --- | --- | --- | --- | --- | --- | --- | --- | --- | --- |
|  |  | [C_t_] | [C_t_] | [C_t_] | [±C_t_] | [% C_t_] |  |  | [r] | SD |
| *28S* | 18.57 | 18.57 | 17.67 | 19.10 | 0.32 | 1.73 | 0.350 | 0.155 | *SDHB* | *28S* |
| *16S* | 17.41 | 17.42 | 16.37 | 19.12 | 0.49 | 2.82 | 0.852 | 0.001 | *16S* | *EF1A* |
| *Tublin* | 21.94 | 21.95 | 20.76 | 23.33 | 0.53 | 2.41 | 0.606 | 0.008 | *18S* | *NADH* |
| *NADH* | 24.18 | 24.18 | 23.31 | 24.93 | 0.47 | 1.93 | 0.680 | 0.002 | *v-ATPase* | *16S* |
| *Actin* | 18.30 | 18.31 | 17.24 | 19.52 | 0.59 | 3.22 | 0.715 | 0.001 | *PRL12* | *SDHB* |
| *TATA* | 26.04 | 26.05 | 24.74 | 28.19 | 0.59 | 2.27 | 0.330 | 0.181 | *Actin* | *PRL12* |
| *PRL12* | 22.22 | 22.22 | 21.40 | 23.19 | 0.50 | 2.27 | 0.742 | 0.001 | *NADH* | *Tublin* |
| *18S* | 10.70 | 10.72 | 9.89 | 11.89 | 0.54 | 5.03 | 0.800 | 0.001 | *Tublin* | *18S* |
| *EF1 A* | 18.47 | 18.48 | 17.87 | 19.70 | 0.37 | 2.01 | 0.442 | 0.066 | *EF1A* | *Actin* |
| *v-ATPase* | 23.66 | 23.68 | 22.63 | 25.91 | 0.80 | 3.39 | 0.781 | 0.001 | *28S* | *TATA* |
| *SDHB* | 22.90 | 22.91 | 22.08 | 23.99 | 0.49 | 2.15 | 0.931 | 0.001 | *TATA* | *v-ATPase* |
